# Mobile Robotic Balance Assistant (MRBA): a gait assistive and fall intervention robot for daily living

**DOI:** 10.1186/s12984-023-01149-0

**Published:** 2023-03-01

**Authors:** Lei Li, Ming Jeat Foo, Jiaye Chen, Kuan Yuee Tan, Jiaying Cai, Rohini Swaminathan, Karen Sui Geok Chua, Seng Kwee Wee, Christopher Wee Keong Kuah, Huiting Zhuo, Wei Tech Ang

**Affiliations:** 1 Rehabilitation Research Institute of Singapore, Nanyang Technological University, Singapore, Singapore; 2 Centre for Rehabilitation Excellence (CORE), Tan Tock Seng Hospital, Singapore, Singapore

**Keywords:** Robotics, Human balance, Gait assistance, Fall intervention

## Abstract

**Background:**

Aging degrades the balance and locomotion ability due to frailty and pathological conditions. This demands balance rehabilitation and assistive technologies that help the affected population to regain mobility, independence, and improve their quality of life. While many overground gait rehabilitation and assistive robots exist in the market, none are designed to be used at home or in community settings.

**Methods:**

A device named Mobile Robotic Balance Assistant (MRBA) is developed to address this problem. MRBA is a hybrid of a gait assistive robot and a powered wheelchair. When the user is walking around performing activities of daily living, the robot follows the person and provides support at the pelvic area in case of loss of balance. It can also be transformed into a wheelchair if the user wants to sit down or commute. To achieve instability detection, sensory data from the robot are compared with a predefined threshold; a fall is identified if the value exceeds the threshold. The experiments involve both healthy young subjects and an individual with spinal cord injury (SCI). Spatial Parametric Mapping is used to assess the effect of the robot on lower limb joint kinematics during walking. The instability detection algorithm is evaluated by calculating the sensitivity and specificity in identifying normal walking and simulated falls.

**Results:**

When walking with MRBA, the healthy subjects have a lower speed, smaller step length and longer step time. The SCI subject experiences similar changes as well as a decrease in step width that indicates better stability. Both groups of subjects have reduced joint range of motion. By comparing the force sensor measurement with a calibrated threshold, the instability detection algorithm can identify more than 93% of self-induced falls with a false alarm rate of 0%.

**Conclusions:**

While there is still room for improvement in the robot compliance and the instability identification, the study demonstrates the first step in bringing gait assistive technologies into homes. We hope that the robot can encourage the balance-impaired population to engage in more activities of daily living to improve their quality of life. Future research includes recruiting more subjects with balance difficulty to further refine the device functionalities.

**Supplementary Information:**

The online version contains supplementary material available at 10.1186/s12984-023-01149-0.

## Background

Physiologic aging processes, musculoskeletal limitations and neuropathology are common causes of impaired balance. Balance control probably has the greatest impact on activities of daily living (ADLs) independence and gait, because it is a fundamental motor skill and prerequisite to the maintenance of a myriad of postures and mobility [1–3]. A consequence of weakened balance control is fall. Patient falls have been a major issue of concern in geriatric care and rehabilitation worldwide as it is the single most crucial factor in patient injuries [4]. Falls also have psychological impacts in which they cause fear, anxiety, and loss of confidence, resulting in activity avoidance, social isolation and increasing frailty [5]. The occurrence rate of falls among older patients is high in both healthcare facilities and at home.

The idea of an overground gait or balance trainer has been explored by the rehabilitation and assistive robotics community in the past decade, most notably is the KineAssist [6] developed at Northwestern University and commercialized by Kinea Design LLC. A similar technology focusing on Parkinson’s Disease patients, the Robotic Walker for Gait Rehabilitation, has been reported by Mun et al. [7] from National University of Singapore (NUS). Because of the inertia of the robots, the users of both systems experience an alteration in gait strategies, especially in the stages of transiting between standing and walking (i.e. starting and stopping). On the other hand, some products were developed based on a suspended harness system to provide body weight support for overground mobility training [8]. Most devices currently available were designed to be used in healthcare institutions, rather than home- or community-based, thus having a large footprint and low maneuverability.

**Fig. 1:**
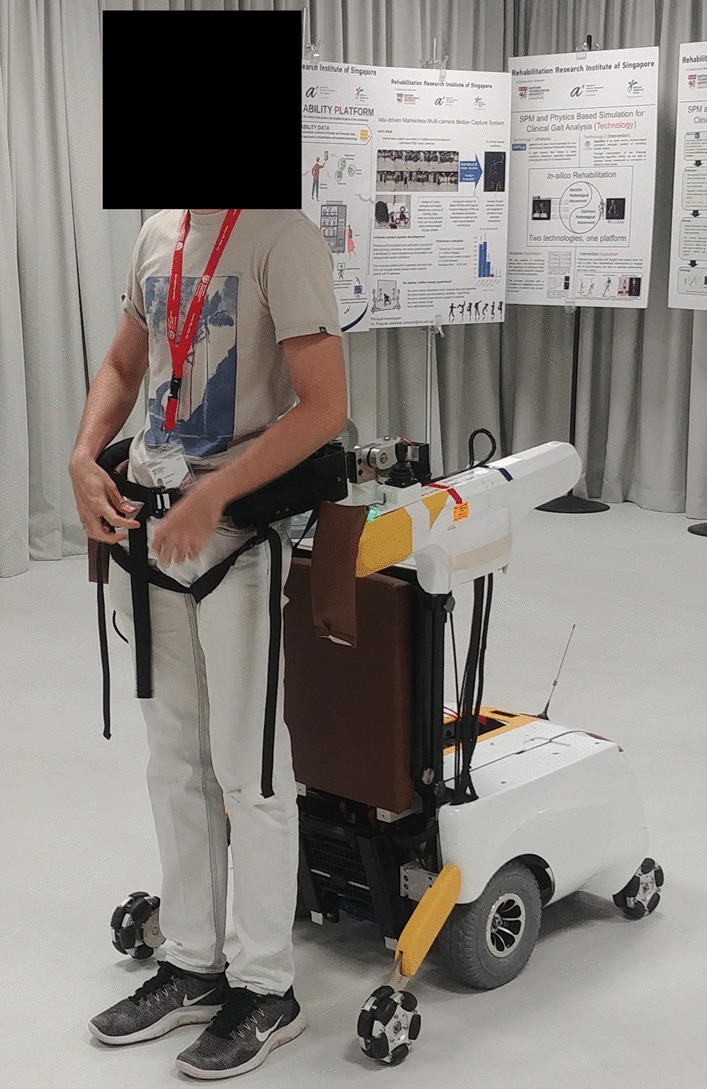
Mobile Robotic Balance Assistant (MRBA) with a user attached to it

A device named Mobile Robotic Balance Assistant (MRBA) was thus developed to address this problem (Fig. 1). The robot provides body weight and balance support to the user during level ground ambulation through its pelvic interface that allows MRBA to track its user autonomously. MRBA follows the user closely while holding onto their pelvis, mimicking the helping hands of a parent when a toddler learns to walk. In case the user loses balance, the robot can intervene the fall by securing the user in place so that they can regain balance. When the user wants to sit down or commute, the robot can be transformed into a powered wheelchair. The motion between sitting and standing are physically supported by the robot. The hybrid design of a gait assistive technology and a wheelchair potentially allows the user to perform a wide variety of ADLs at home and in the community with only one piece of mobility aid.

While the robot is designed for home use, it can also be used in clinical settings to relieve the burden of physiotherapists. It may also help in rehabilitation as it encourages the user to engage in walking and ADLs by providing added safety features. Such home- and community-based rehabilitation is a complement to hospital-based rehabilitation and may promote better recovery in motor capability as it increases the training dose by allowing patient to practice more walking and balancing tasks. Nevertheless, as patients’ mobility and independence are encouraged in the paradigm, the risk of falls is further increased [9–12]. Notwithstanding, having a device like MRBA ensuring the safety of the user allows the user to participate in a more active lifestyle.

## Methods

### Hardware design

**Fig. 2:**
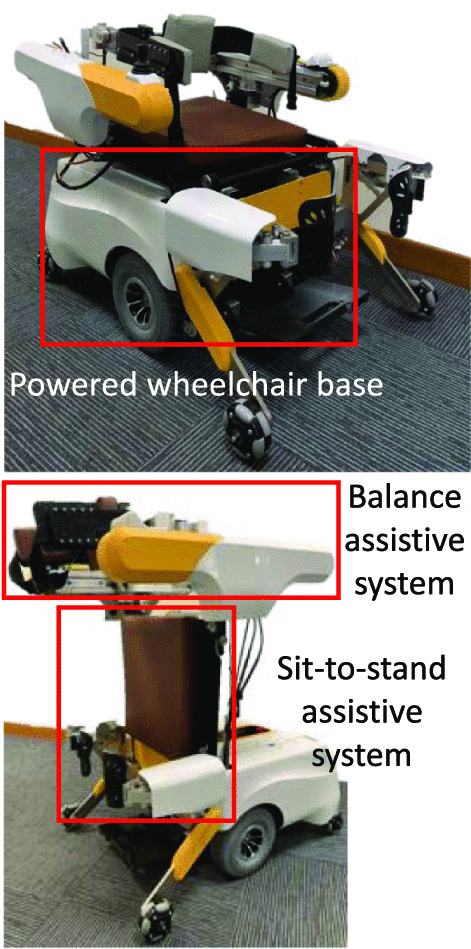
Overview of Mobile Robotic Balance Assistance. Top: Wheelchair configuration; Bottom: Walking-assisted configuration

MRBA consists of four sub-systems: namely (i) a powered wheelchair base, (ii) a sit-to-stand assistive system, (iii) a balance assistive system, and (iv) the sensory system (Fig. 2). They allow the robot to assist the user’s mobility in both Wheelchair Configuration and Walking-assisted Configuration.

#### Powered wheelchair base

When transformed into the Wheelchair Configuration, the device serves as a powered wheelchair that allows the user to commute quickly. The mobile base was built with a footprint of 1.05 m $\times$ 0.7 m, which is comparable to regular wheelchairs, making it small enough to navigate in the home environment. The flexibility is further enhanced by the mid-wheel driven design, which leads to a small turning radius of 65 cm. The two driving wheels are driven by DC Motor (EC82N245325ALGB, Motion Tech Motors), whereas the four anti-tip omni wheels (127 mm Heavy Duty Aluminum, Nexus Automation) help to stabilize the platform in case of a loss of balance. The robot has a maximum speed of 2.2 m/s, which is sufficient to move along with a balance-challenged user, whose walking speed is often below 1.0 m/s [13–15].

#### Sit-to-stand assistive system

**Fig. 3:**
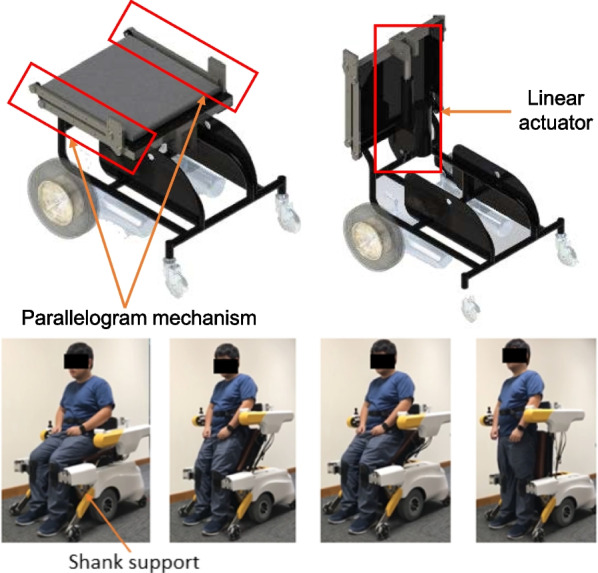
The sit-to-stand mechanism. Top left: Wheelchair configuration; Top right: Walking-assisted configuration. Bottom: The process from sit to stand

The sit-to-stand mechanism consists of two parts: a linear actuator (LX700S100, LYX) and a customized parallelogram mechanism. They together form a four-bar linkage, as shown in Fig. 3. When the linear actuator extends, the mechanism lifts the seat from the horizontal orientation to the vertical orientation, assisting the user when they are standing up; when the linear actuator contracts, the mechanism folds the seat such that the user can sit down at a safe pace. Throughout the process, the parallelogram maintains the orientation of the balance assistive mechanism such that it is always horizontal with respect to the ground.

**Fig. 4:**
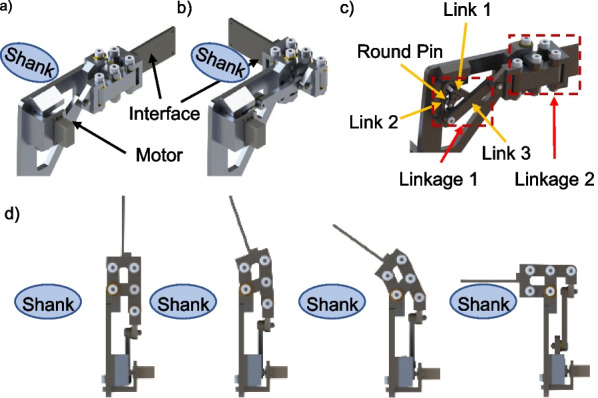
The shank support mechanism. **a** Fully open configuration. **b** Fully closed configuration. **c** Linkages without the motor. **d** Shank support operates from open to closed configuration

To prevent the user from falling forward during the sit-to-stand process, a shank support mechanism was designed. The mechanism is actuated to open and close the shank holding interface as shown in Fig. 4a, b, d. The mechanism is closed before the user starts to stand such that the interface comes into contact with the shank to prevent the lower limbs from buckling. The mechanism then opens after the user stands straight so that it does not hinder the walking movements. The details of the mechanism are illustrated in Fig. 4c with the motor hidden. There are two linkages in the mechanism. In Linkage 1, the motor drives Link 1 and transmits the movement from Link 1 through Link 2 to Link 3. Link 1 connects to Link 2 through a round pin and slot whereas Link 2 connects to Link 3 through a revolute joint. Link 3 moves Linkage 2, which is a four-bar mechanism, to perform the open and close actions.

The entire sit-to-stand assistance process, including the seat lifting and shank supporting, takes approximately 15 s.

#### Balance assistive system

The balance assistive system consists of the pelvic interface, the brakes and the force transmitting cables.

**Fig. 5:**
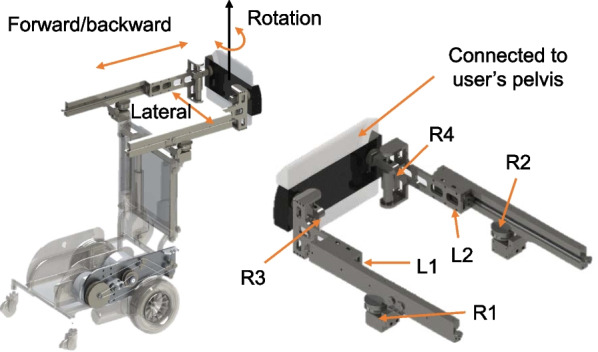
The pelvic interface. It connects the robot to the user at the pelvic area such that the pelvis can move in three degrees of freedom on the horizontal plane

The interface is a pair of robotic arms formed by four rotary joints (R1–R4) and two linear joints (L1, L2), as shown in Fig. 5. It is attached to the user at the pelvis and thighs via a belt and a harness support, respectively. The contact point is chosen to be at the pelvic area because it is close to the Center of Mass (CoM) of the human and thus allows the assistive forces to be delivered more effectively. Unlike other gait assistive devices that use rigid interface coupled with force sensors to follow the user’s motion via feedback control, MRBA interface is intrinsically compliant due to its rotary and prismatic joints. Thus, the interface needs not to be directly actuated for compliant interaction. The compliant property decouples the dynamics of the mobile base from the user, reducing the inertia effect of the mobile platform on the user. In total, the interface provides three degrees of freedom that caters for natural pelvic movement along the horizontal plane. The range of motion is indicated as follows:

- Anterior–Posterior: − 200 mm–200 mm
- Lateral: − 150 mm–150 mm
- Rotation: − 60$$^\circ$$–60$$^\circ$$

In the recent years, variable stiffness actuators or mechanisms have been widely adopted in robotic systems with direct human contact. The controllable compliance reduces the impact on human body as a portion of the interaction force or torque is absorbed by the spring and damper system in the mechanism. However, such systems demand complex control algorithms and expensive high-power motors. Instead, MRBA utilizes magnetic powder brakes (TJ-POD-1.5, Tian Ji) to modulate the stiffness of the pelvic interface. The biggest advantage of using magnetic powder brake is the linear relationship between the input voltage and the brake force, making it easy for stiffness adjustment. The system then resists the interface movement in unwanted direction such that the CoM remains in the safe zone. If the user loses balance, the interface can be stiffened almost instantaneously to arrest the fall; the pelvic interface can recover its compliance after the user regains their balance.

**Fig. 6:**
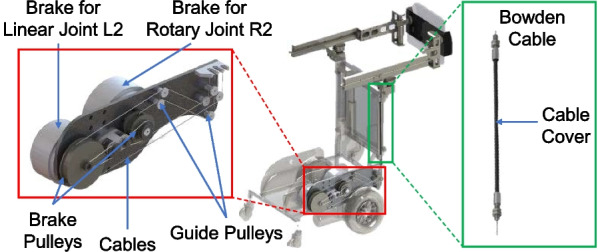
The variable stiffness actuation system with Bowden cables

However, magnetic powder brakes are usually bulky, making it impossible to directly couple the brakes to the robotic arm joints. Thus, a novel cable driven mechanism was developed to transmit the forces from the actuators to the joints (see Fig. 6). With the new design, the heavy brakes can be installed on the mobile base, greatly reducing the weight of the robotic arms such that the compliance of the pelvic interface is maintained. Bowden cable is used for its flexibility as it can adapt its shape to both sitting and standing configurations. A cable routing system is used to guide the cables from the Bowden cable ends to the brakes. The system contains two types of pulleys: the brake pulleys that are directly connected to the brakes, and the guide pulleys that guides the cable from the brake pulleys to the Bowden end connectors. The four variable stiffness brakes are connected to the joints R1, R2, L1 and L2 of the pelvic interface through the cable driven mechanism. The mechanical response time of the fall intervention system is 0.28 s.

The entire system weighs 140 kg and is able to provide balance support to patients weigh up to 80 kg.

#### Sensory system

The intended function of the system is to mimic a therapist assisting patient during overground walking training. Thus, the robot must be able to identify the patients’ movements and balance state to follow the patient walking and provide balance assistance when necessary. Four potentiometers (WDD35D-4, Wenzhou Xinle Instrument and Meter Co., Ltd) are installed to measure the motion of joints L1, L2, R3, R4 as labeled in Fig. 5. They are used to determine the position and the orientation of the pelvic interface. As the interface is directly connected to the human pelvis, we can monitor the user pelvic movement and use this as the target to follow the user.

Evaluating the human balance state is a much more challenging task. The literature have suggested monitoring the trunk motion to identify one’s instability right before a fall impact [16–20]. Hence, an Inertial Measurement Unit (IMU) (BN0055, BOSCH) is placed on the pelvic interface to monitor the kinematics of the subject’s CoM. Meanwhile, as the user loses balance, part of their body weight will be supported by the robotic arm. Thus, as another balance evaluation module, a couple of force sensors (ZNHM-7-500 KG, Chino Sensor) are installed at each side of the robotic arm to measure the vertical forces exerted on the robot.

**Fig. 7:**
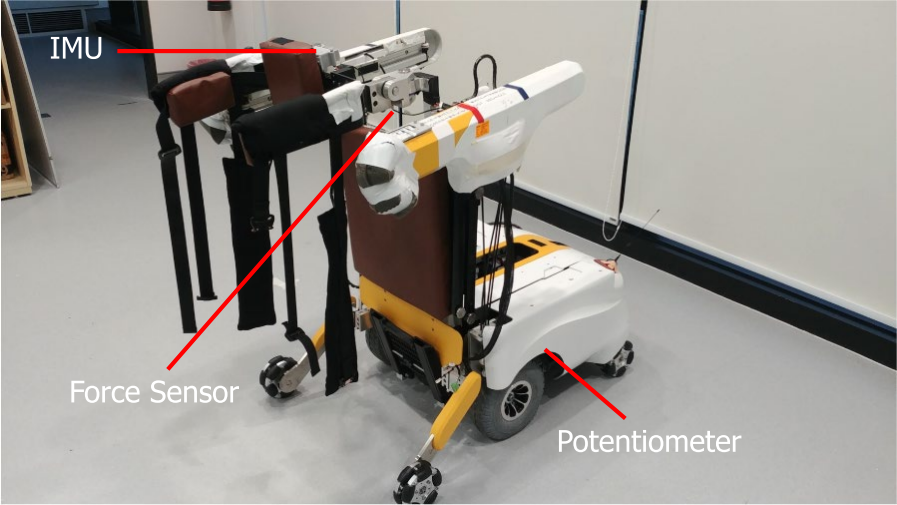
Sensor locations on MRBA

Figure 7 illustrates the sensory components on the robot. The major components of MRBA are summarized in Table 1.

**Table 1:** List of major components of MRBA

Components	Manufacturer	Model No.
Driving Wheel Motors	Motion Tech Motors	EC82N245325ALGB
Anti-tip Omni Wheels	Nexus Automation	127mm Heavy Duty Aluminum
Linear Actuator	LYX	LX700S100
Magnetic Brakes	Tian Ji	TJ-POD-1.5
Potentiometers	Wenzhou Xinle Instrument and Meter	WDD35D-4
Force Sensors	Chino Sensor	TJZNHM-7-500KG
IMU	BOSCH	BN0055

### Control architecture

MRBA runs three modes of operation:

- Walking Assisted Mode: MRBA is in the Walking-assisted configuration. It follows the user movement as they walk around in the environment. In case of fall, assistance will be provided to the user to intervene the fall. Then, the user can recover by themselves or call for assistance.
- Sitting Mode: MRBA is in the Wheelchair Configuration. It serves as a powered wheelchair.
- Transient Mode: MRBA transforms between the Wheelchair Configuration and the Walking-assisted Configuration, and vice versa. The user receives physical support during the standing up and sitting down motion.

The remaining of the section discusses the user following algorithm and fall intervention strategy implemented in the Walking Assisted Mode.

#### User following algorithm

**Fig. 8:**
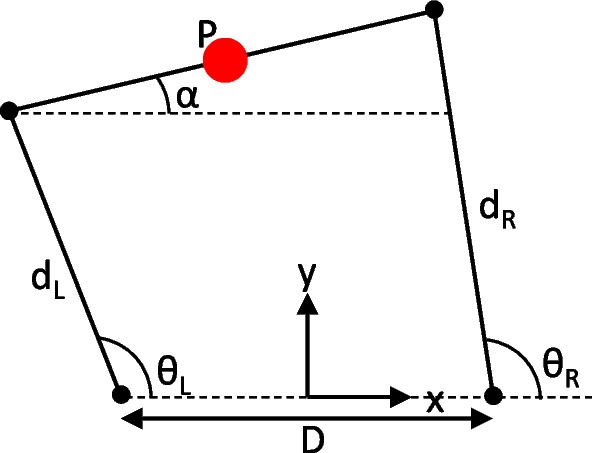
Geometry of the pelvic interface. *P* is the center of the pelvic interface, $\alpha$ is the facing orientation of the interface, $d_L, d_R, \theta _L, \theta _R$ are the distances of the left and right sliders from the rotary joints, and the angles of the left and right arms with respect to the ML-axis, respectively, *D* is the distance between R1 and R2 of the two robotic arms

MRBA follows the user when they walk around by tracking the state of the person’s CoM with respect to the robot. The CoM position and facing orientation of the subject is assumed to be aligned with the pelvic interface (Fig. 8):

1 $$\begin{aligned} x_P= & {} \frac{d_L\cos (\theta _L)+d_R\cos (\theta _R)}{2} \end{aligned}$$

2 $$\begin{aligned} y_P= & {} \frac{d_L\sin (\theta _L)+d_R\sin (\theta _R)}{2} \end{aligned}$$

3 $$\begin{aligned} \alpha= & {} \arcsin \left( \frac{d_L\sin (\theta _R)-d_R\sin (\theta _L)}{D}\right) \end{aligned}$$

where $(x_P,y_P)$ are the CoM coordinates, $\alpha$ is the facing orientation of the interface, $d_L, d_R, \theta _L, \theta _R$ are the distances of the left and right sliders from the rotary joints, and the angles of the left and right arms with respect to the medial–lateral (ML) axis, respectively, *D* is the distance between the joints of the two robotic arms.

**Fig. 9:**
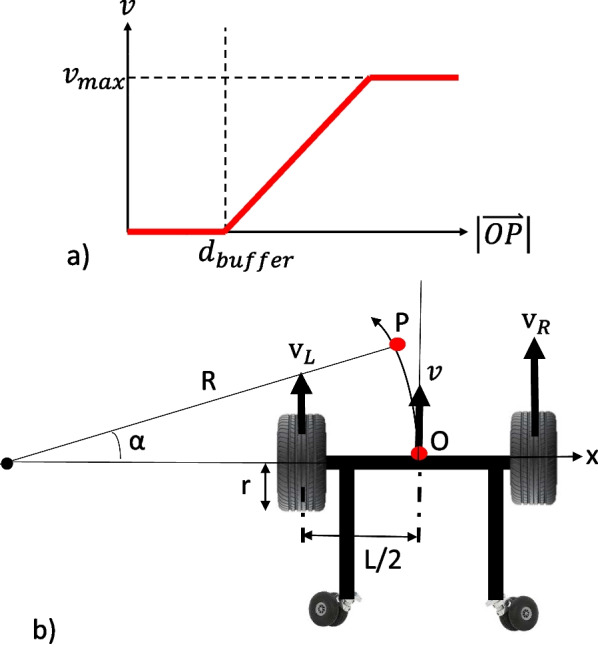
**a** The plot of the MRBA speed against the distance between the user CoM and MRBA. **b** Illustration of wheelchair base motion, where *R* is the turning radius, *r* is the wheel radius, *L* is the distance between the wheels, $v, v_L, v_R$ are the speeds of the CoM, left wheel and right wheel, respectively

The principle of the User Following Algorithm (Fig. 9a) is to maintain a safe distance between the user and the robot. If the distance of the CoM with respect to the robot $|\vec {OP}|$ exceeds some predefined threshold $d_{buffer}$, the user is assumed to be moving towards the direction of the CoM. The robot will move towards the user to catch up with them. The maximum robot speed $v_{max}$ is set to be the average human walking speed of 1.2 m/s [21]. The wheelchair also minimizes the angle between the human pelvis and the mobile base to align its heading direction with the user’s.

Due to the non-omnidirectional nature of the robot, it moves with a turning radius *R* when $x_P \ne 0$, depending on the speeds of the wheels. From Fig. 9b, the turning radius of the robot is given to be:

4 $$\begin{aligned} R = \frac{|\vec {OP}|}{2\sin (\frac{\alpha }{2})} \end{aligned}$$

The desired CoM speed *v* is set to be proportional to the arc $\overset\frown{\text{OP}}$, i.e. $v=k\overset\frown{\text{OP}}$, where *k* is a positive scalar. The respective wheel speeds can then be computed as:

5 $$\begin{aligned} \omega _R = \frac{k\alpha (R+\frac{L}{2})}{r} \qquad \omega _L = \frac{k\alpha (R-\frac{L}{2})}{r} \end{aligned}$$

where *r* is the wheel radius.

**Fig. 10:**
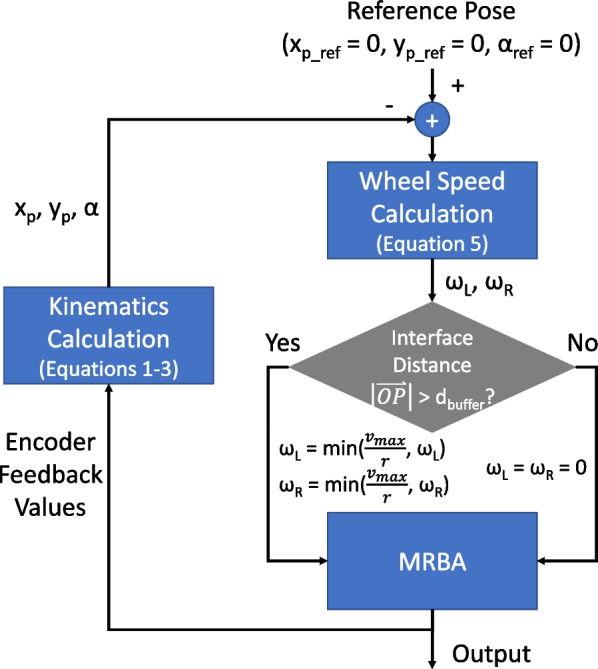
The control block diagram of the User Following Algorithm

The control block diagram of the User Following Algorithm is illustrated in Fig. 10.

#### Instability detection and fall intervention

One of the main objectives of MRBA is to improve human safety by intervening falls in vulnerable subjects. When a patient is performing ADLs with MRBA, they may lose balance due to their weakened balance control. To safeguard the user’s safety, an instability detection and fall intervention system is critical. As a preliminary approach, the force sensors and the IMU are used to identify instability. The forces exerted by the user onto the robot are monitored through the force sensors. If the force magnitude exceeds a certain threshold, the user is deemed to have lost their balance. For the case of IMU, any increase in the acceleration or gyroscope measurements that exceeds predefined thresholds will be treated as a loss of balance. When instability is detected, the magnetic brakes connected to the robotic arms joints will be activated to restrict any interface motion. The mobile base will also come to a halt. This holds the user in place so that they can regain balance by themselves or wait for help to arrive.

**Fig. 11:**
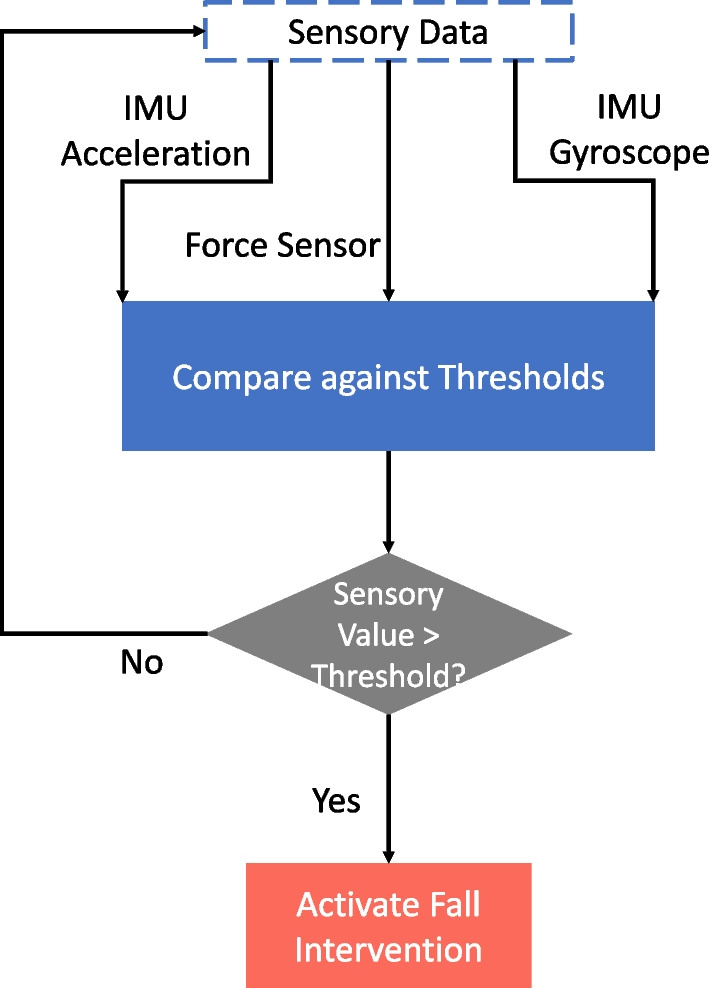
The control block diagram of the Instability Detection Algorithm. Each sensory data is compared to their corresponding thresholds

The control block diagram of the Instability Detection Algorithm is illustrated in Fig. 11.

### Experimental protocols

Experiments were designed to evaluate the performances of the User Following Algorithm and the Instability Detection Algorithm.

An ideal gait assistive device should be completely transparent to the user in which it does not interfere with their movement unless a loss of balance is detected. Notwithstanding, complete compliance is difficult to achieve, especially when the pelvic interface of MRBA is physically connected to the user to safeguard their stability. A compliant User Following Method can help to alleviate the hardware impedance to the user movement. The User Following Algorithm can thus be evaluated indirectly through the effect of MRBA on human gait by comparing the user’s gait when walking with and without the robot.

The Instability Detection Algorithm can be assessed using normal walking trials and simulated falls. The Algorithm should identify the loss of balance induced during the falls but not produce any false alarms when the user is walking normally. To induce instability on the subjects, a device named Fall Inducing Movable Platform (FIMP) was used to provide ecologically valid, systematic, and reproducible falls [22]. The system consists of a metal frame in which the person walks; an RGB-D camera on the platform allows it to follow the subject’s movement by maintaining a fixed distance from the subject. The subject’s lower limb is connected to the device, which is actuated to perturb the motion of the lower limb and cause the subject to lose their balance.

The experiment involved the following actions.

- Free Walking (**FW**): The subject walked in a straight line for 7 m at their preferred speed. The action was repeated four times.
- Normal Walking with MRBA
  - Straight Walking (**NW_SW**): The subject walked in a straight line for 7 m at their preferred speed. The action was repeated four times.
  - Walking in Circles (**NW_C**): The subject walked in circles within a rectangular area of 5 m $\times$ 1.5 m at their preferred speed. The subject walked in the clockwise directions for two laps. The same protocol was conducted for anti-clockwise direction.
- FIMP-induced Falls with MRBA
  - Mid Swing Trip (**FE_MS**): The subject’s left ankle was connected to the electrical brake of FIMP. The subject walked in a straight line. After a few steps, the brake activated during the left mid-swing to prevent the foot from moving forward. The action simulated a mid-swing trip in which the foot hits an obstacle during the swing phase. The action was repeated at least six times.
  - Slip (**FE_SL**): The subject’s left ankle was connected to the electrical motor of FIMP. The subject walked in a straight line. After a few steps, the motor activated during the left heel strike and pulled the foot forward. To increase the effectiveness, the subject walked on sliding sheets. The action simulated a slip on a slippery surface. The action was repeated at least six times.
  - Knee Buckling (**FE_KB**): The subject’s left knee was connected to the electrical motor of FIMP. The subject walked in a straight line. After a few steps, the motor activated during left mid-stance and pulled the knee forward. The action simulated knee muscle weakness. The action was repeated at least six times.

During the FIMP-induced trials, the subjects were instructed to regain balance by themselves if possible. However, to ensure the subjects’ safety, they were allowed to press an emergency button when they required support from the robot. This would trigger the fall intervention function of MRBA, causing it to stop completely and hold the subject in place.

Twelve healthy young subjects (four females, age: 27.3 $\pm$ 3.2 years of age, height: 1.70 $\pm$ 0.06 m, weight: 63.6 $\pm$ 8.9 kg) were recruited. In addition, to understand the effect on pathological gait, a subject with walking and balance disability (female, age: 54 years of age, height: 1.54 m, weight: 66.3 kg) was recruited as a preliminary study. The subject suffered from an incomplete spinal cord injury (SCI) at L4/5 level 25 years ago, resulting in Grade C ASIA Impairment Scale. She had no prior experience with rehabilitation and assistive robots. Due to safety concerns, the SCI subject only performed the FW and NW_SW trials.

Nonetheless, the first experiment (Fall Experiment 1) revealed the limitations of the FIMP-induced falls (to be discussed in subsequent Sections). Hence, a new experiment was proposed to introduce subject-induced falls to further examine the instability detection algorithm. The second experiment (Fall Experiment 2) involved the following actions:

- Normal Walking with MRBA
  - Straight Walking (**NW_SW**).
  - Walking in Circles (**NW_C**).
- Self-induced Falls with MRBA
  - Reaching Out Fall (**FS_RO**): The subject stood and reached outwards to retrieve an object until lost balance. The action was repeated at least six times.
  - Walking and Fall (**FS_W**): The subject walked in a straight line normally for a few steps and fell at heel strike to simulate muscle weakness at lower limb. The action was repeated at least six times.

Eight healthy young subjects (three females, age: 28.4 $\pm$ 4.6 years of age, height: 1.70 $\pm$ 0.10 m, weight: 67.2 $\pm$ 9.1 kg) were recruited for Fall Experiment 2. They were instructed to not recover the fall by themselves unless necessary, in which they can press the emergency button to trigger the fall intervention system.

The MRBA sensory data, namely the acceleration and gyroscope from the IMU, as well as the force sensor measurement were collected for analysis. Infrared markers were placed on the lower body of the subjects according to the CAST lower body marker set [23] such that their movement can be recorded by the motion capture system (Qualisys Miqus M3 (2MP)). The motion capture data were then used to compute the joint angles and the gait parameters using Visual3D v6 Professional by C-motion. The following joint angles were extracted:

1. Pelvic obliquity
2. Hip flexion/extension
3. Knee flexion/extension
4. Ankle dorsi-/plantar-flexion

The gait events, i.e. heel strike and toe off, were labeled manually to segment the data into gait cycles.

To compare the time series data of motion kinematics and sensory values in a continuous manner, statistical parametric mapping (SPM) [24] was used. SPM is commonly applied in biomechanics [25], making it ideal in fall-related studies. A Python library developed for 1-dimensional SPM (SPM1D) [24] was used in this investigation. SPM1D computes the mean and variance curves of two or more datasets, which are then analyzed with linear statistical testing such as t-test and Analysis of Variance (ANOVA). The resultant statistics, known as parametric maps, are processed with random field theory to generate a threshold, from which the statistical significance is determined. When a part of the time series has a significant difference among the datasets, it forms a cluster that exceeds the defined threshold. The p-value of that cluster indicates the probability of the cluster could have resulted from a smooth random process. To implement SPM1D, each sequence of the time series data has to be comparable directly. Hence, the data were time-normalized in terms of gait cycles, i.e. the data in each stride was normalized into stance and swing phase, with the former occupying 60% of the cycle and the latter covering the remaining 40%; the 0% and 100% marks of the gait cycle indicate the heel strike of the stride. In this study, the SPM1D analysis of one-way ANOVA was applied with an alpha threshold of 0.05.

The proposed Instability Detection Algorithm was tested with the sensory data. A trial is considered as detected if the sensory data exceeds the threshold. A fall trial is only considered as a successful detection if the measurement exceeds the threshold after the activation of the FIMP fall-inducing actuators. The performance of the fall detection algorithm is determined from its sensitivity and specificity using G-mean, which is defined as the geometric mean of sensitivity and specificity.

6 $$\begin{aligned} Sensitivity= & {} \frac{\text {number of detected fall trials}}{\text {number of fall trials}} \end{aligned}$$

7 $$\begin{aligned} Specificity= & {} \frac{\text {number of non-detected walking trials}}{\text {number of walking trials}} \end{aligned}$$

8 $$\begin{aligned} G\text {-}mean= & {} \sqrt{Sensitivity \times Specificity} \end{aligned}$$

For each algorithm, different thresholds were tested to find the one that gave the largest G-mean score, i.e. identified the most cases of instability while neglecting the normal walking trials.

**Fig. 12:**
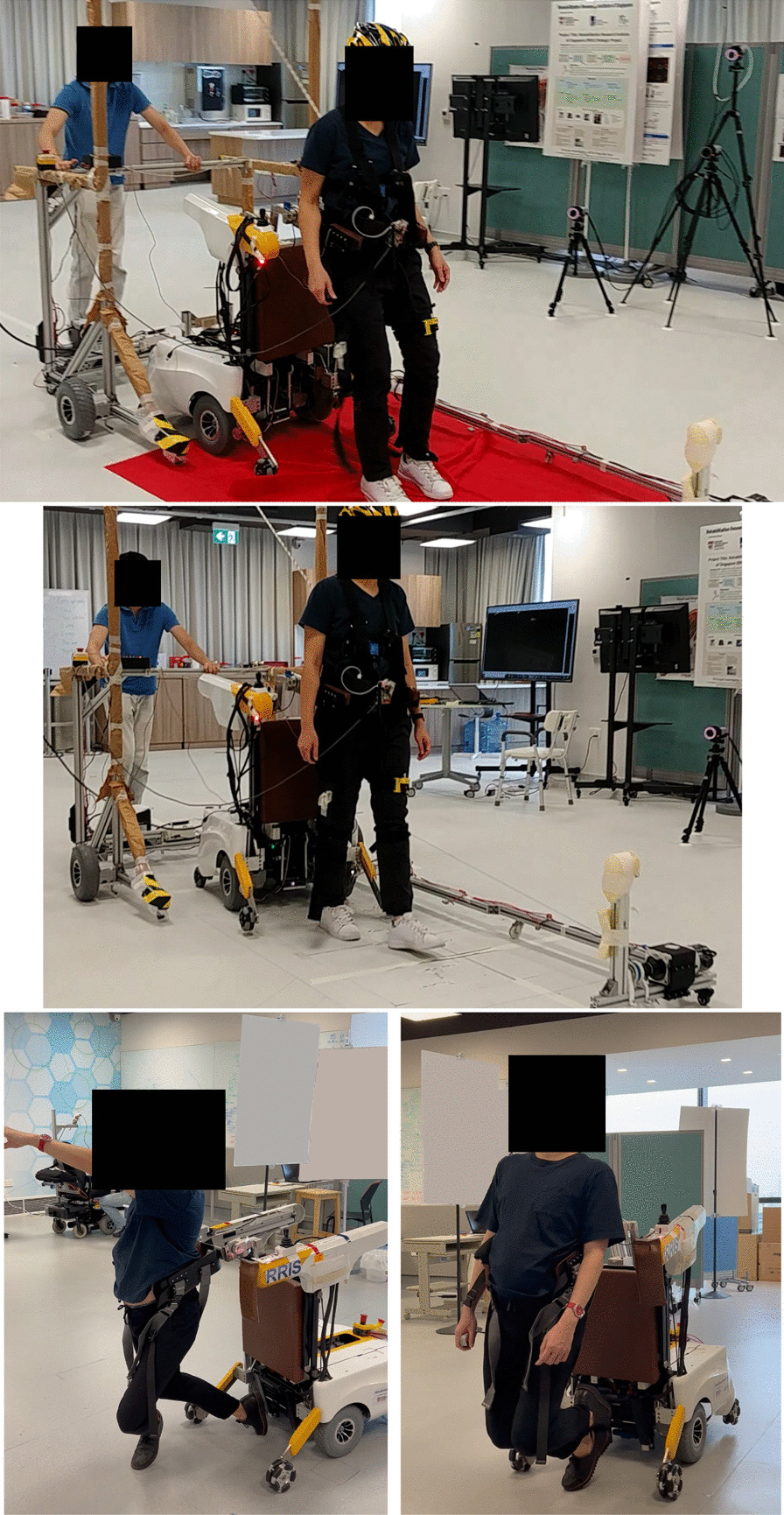
Top: An FE_SL trial that involved FIMP to trigger slip by pulling the subject’s left ankle forward when the left foot struck the sliding sheets. Middle: An FE_KB trial that involved FIMP to trigger knee buckling by pulling the subject’s left knee forward during left mid-stance. Bottom left: An FS_RO trial in which the subject fell when reaching out to grab an object. Bottom right: An FS_W trial in which the subject fell when walking

The experiment protocols were approved by the Institutional Review Board of Nanyang Technological University, Singapore (Application ID: IRB-2019-09-028 and IRB-2021-050). A few pictures taken during the experiment are shown in Fig. 12. The reader is referred to Additional file 1 for a demonstration video of MRBA.

## Results

The recorded data were screened for recording quality and instability inducing accuracy. In Fall Experiment 1, 48 FW, 52 NW_SW, 24 NW_C, 31 FE_MS, 51 FE_SL and 35 FE_KB trials from the healthy subjects, and 4 FW and 4 NW_SW trials from the SCI subject were used in the analysis. In Fall Experiment 2, 32 NW_SW, 16 NW_C, 48 FS_RO and 48 FS_W were used.

### User following algorithm

The effect of MRBA on human gait can be demonstrated through the healthy subjects’ gait parameters during FW and NW_SW, which is tabulated in Table 2. Student’s t-test was performed to compute the statistical significance between the two datasets. A significant decrease in speed was observed as the robot was unable to follow the user smoothly at higher gait speed. The robot also forced the subjects to take smaller steps for it to follow the user more smoothly. The step width of the subjects remained constant while the step time increased slightly.

**Table 2:** The gait parameters (mean $\pm$ standard deviation) of healthy subjects (n=12) during free walking (FW) and walking with MRBA (NW_SW) in a straight line

Gait parameters	FW	NW_SW	p-values
Speed (m/s)	1.16±0.19	0.76±0.15	< 0.001
Step length (m)	0.66±0.09	0.46±0.08	< 0.001
Step width (m)	0.13±0.03	0.13±0.04	0.364
Step time (s)	0.58±0.05	0.62±0.07	< 0.001

The data is compared with a Student’s t-test

**Fig. 13:**
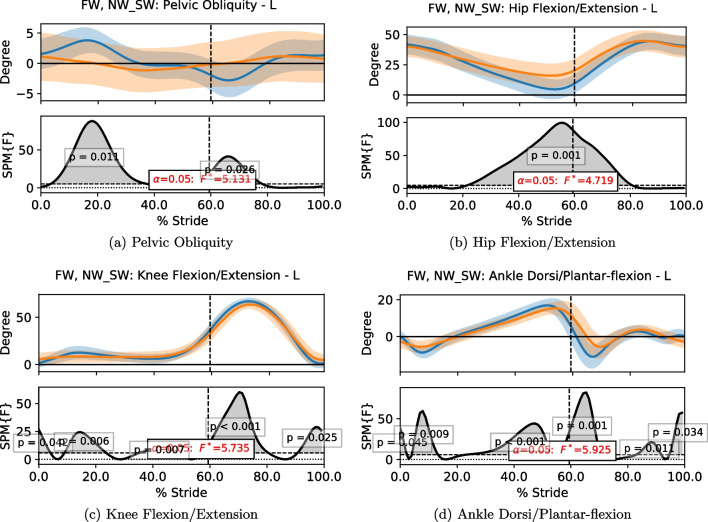
The plots of the data trend of body joint angles and their SPM1D results of free walking (FW) and walking with MRBA (NW_SW) for healthy subjects. The blue line and the orange line represent the data of FW and NW_SW, respectively. The dotted line represents the end of the stance phase and the beginning of the swing phase. In each subfigure, the first row shows the mean of the data with its standard deviation as shaded region. The second row shows the F-values compared against the threshold. Statistical results greater than the threshold indicate a statistically significant difference between the two groups

The joint angles comparison is given in Fig. 13, which illustrates that the joint angles were altered significantly during MRBA walking. The results show that the subjects had smaller hip joint range of movement, smaller knee flexion and smaller ankle plantar-flexion due to the smaller steps taken. The variation in pelvic movement was also dampened by the robot interface.

**Fig. 14:**
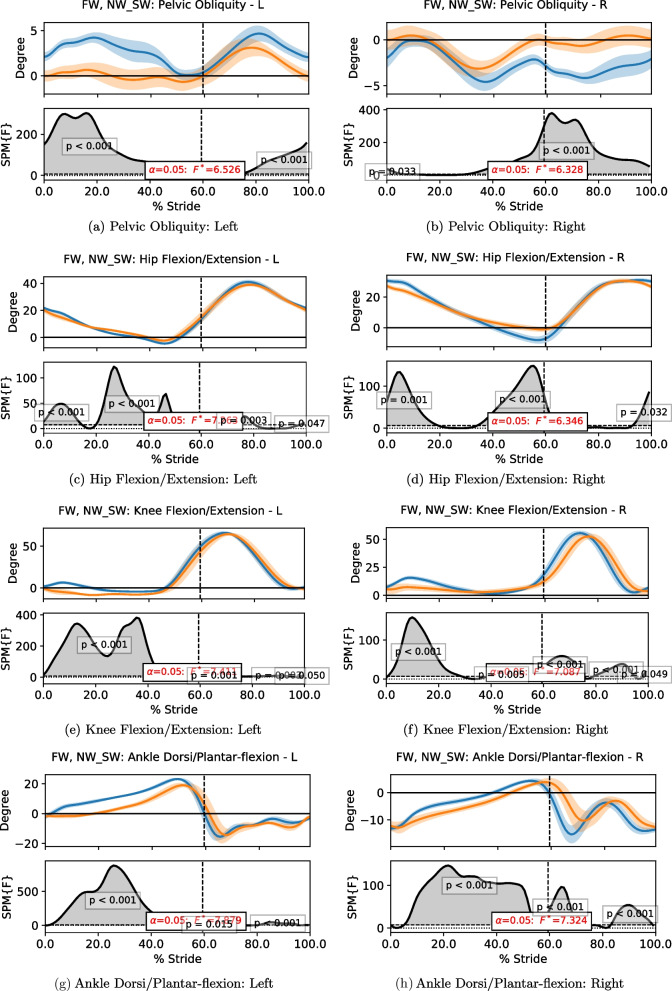
The plots of the data trend of body joint angles and their SPM1D results of free walking (FW) and walking with MRBA (NW_SW) for a subject with SCI. The blue line and the orange line represent the data of FW and NW_SW, respectively. The dotted line represents the end of the stance phase and the beginning of the swing phase. In each subfigure, the first row shows the mean of the data with its standard deviation as shaded region. The second row shows the F-values compared against the threshold. Statistical results greater than the threshold indicate a statistically significant difference between the two groups

The results from the SCI subject are presented in Table 3 and Fig. 14. One may expect that the subject would not experience any gait speed change as her preferred speed of 0.70 m/s was comparable to the NW_SW speed of the healthy subjects. Nonetheless, MRBA still slowed her down such that she walked at 0.48 m/s with the robot. Like the healthy individuals, the step length decreased as she was forced to take smaller steps. However, the decreased step width implied that the subject was more stable when being supported by MRBA. Her step time increases slightly from 0.62 s to 0.72 s. The robot affected her joint kinematics more significantly when compared with healthy individuals.

**Table 3:** The gait parameters (mean $\pm$ standard deviation) of a subject with SCI during free walking (FW) and walking with MRBA (NW_SW) in a straight line

Gait Parameters	FW	NW_SW	p-values
Speed (m/s)	0.70±0.04	0.48±0.04	< 0.001
Step Length (m)	0.43±0.05	0.33±0.04	< 0.001
Step Width (m)	0.25±0.05	0.07±0.03	< 0.001
Step Time (s)	0.62±0.08	0.72±0.10	< 0.001

The data is compared with a Student’s t-test

### Instability detection algorithm

MRBA sensory data from Fall Experiment 1 were analyzed to understand how the measurements varied during straight walking, turning and a loss of balance. To compare the measurements from different trials, the data were segmented according to the labeled gait events as per previous section. For NW_C, only the portion when the person was turning was extracted. For the FIMP-related trials, only the gait cycles containing the FIMP actuator triggering time were included. As the left foot did not always take a new step after the slip and knee buckling, the left foot gait cycles for FE_SL and FE_KB are omitted. The sensory data from the relevant gait cycles were analyzed with SPM1D. See Additional files 2, 3, 4 and 5 for the complete plots of the data trend comparing NW_SW with NW_C, FE_MS, FE_SL and FE_KB, respectively.

**Fig. 15:**
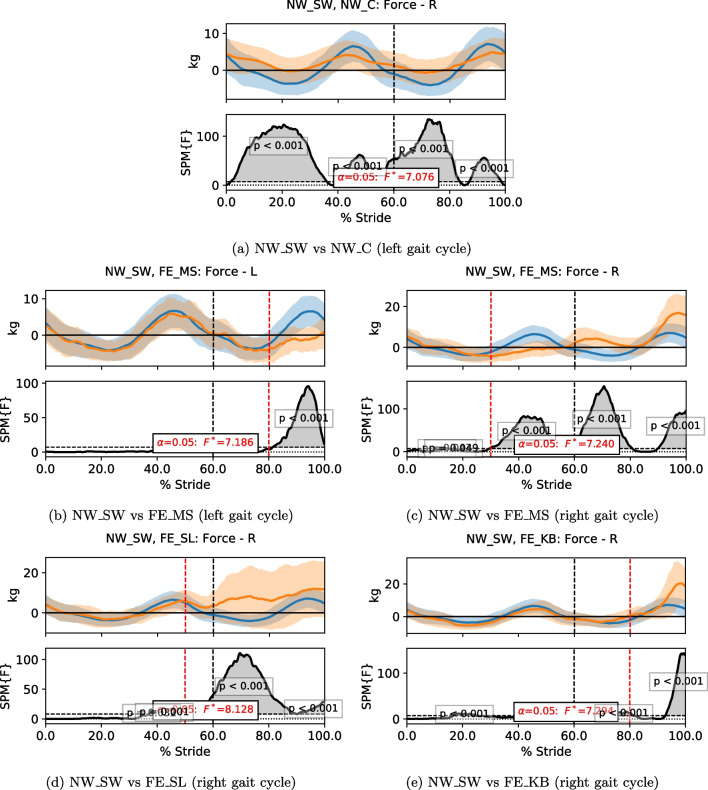
The plots of the data trend of force measurement and their SPM1D results when comparing NW_SW with other trials. The blue line and orange line represent the data of NW_SW and the respective walking trials, respectively. The dotted line represents the end of the stance phase and the beginning of the swing phase while the red dotted line is the fall triggering instance. In each subfigure, the first row shows the mean of the data with its standard deviation as shaded region. The second row shows the F-values compared against the threshold. Statistical results greater than the threshold indicate a statistically significant difference between the two groups. Only one side of the NW_C gait cycle is shown due to symmetry. As the left foot did not always take a step after the slip and knee buckling, the left foot gait cycles are omitted

For acceleration data, the vibration of the robot was captured by the IMU, resulting in a noisy measurement. Nonetheless, during a fall, a sharp peak could be seen if the loss of balance was severe. The turning motion in the NW_C trials causes the gyroscope readings (GYRO) along the anterior–posterior (AP) and vertical axes to have a larger spread. In the fall trials, GYRO along the ML axis went out of phase with NW_SW after FIMP was triggered. In some cases of loss of balance, a sharp peak in the vertical GYRO could be seen. During a natural walking gait, the CoM oscillates along the vertical axis; the CoM moves downward when approaching heel strike and moves upward after heel strike. Such motion will exert forces onto the pelvic interface, causing a minor change in the force data measured by the sensor, as shown in Fig. 15. The forces were smaller in magnitude during turning, presumably due to the subjects walking more cautiously, resulting in milder motions. After the fall-induced actuators were triggered, the force exerted on the interface increased as the subjects lost balance. The SPM1D results show that the force profiles changed differently among the various types of walking trials.

According to the data collected from Fall Experiment 1, the results of the best three sensory data are shown in Table 4.

**Table 4:** The results of instability detection methods in Fall Experiment 1

Methods	NW_SW Detection Rate	NW_C Detection Rate	FE_MS Detection Rate	FE_SL Detection Rate	FE_KB Detection Rate	Sensitivity	Specificity	G-Mean
Force	0.212	0.292	0.645	0.667	0.714	0.675	0.763	0.717
Gyroscope (lateral)	0.615	0.750	0.581	0.471	0.886	0.624	0.342	0.462
Acceleration (anterior–posterior)	0.385	0.500	0.387	0.216	0.343	0.293	0.578	0.416

Detection rate $$= \frac{\text {number of detected trials}}{\text {number of trials}}$$

The force sensor data is the best feature to be used with a G-mean score of 0.717, which is 1.5 times the one of the second-best feature. It is able to detect more than two-thirds of the loss of balance trials, with the highest detection rate of 0.714 in the FE_KB trials. Nonetheless, the false positive rate is higher than 20% with many normal walking trials being wrongly identified as a loss of balance. The IMU features perform poorly in instability detection, presumably due to the noise present in the data and the minimal difference between normal walking and loss of balance. Comparing GYRO along the lateral axis with a threshold, the algorithm achieves a 0.88 detection rate for FE_KB. However, the high true positive rate comes at a cost of a large number of false alarms, which severely impact the usability of MRBA as a product. On the other hand, the anterior–posterior acceleration data yields mediocre specificity but poor sensitivity due to a large number of undetected loss of balance; less than 40% of the FIMP-induced falls were identified when using the feature. The low G-mean scores of the IMU data shows the challenges of utilizing these features for instability detection.

Observing the behavior of the subjects in Fall Experiment 1, the FIMP had marginal effects on the subjects’ stability due to the high balance capability of the healthy young subjects. Also, the subjects were instructed to regain balance by themselves during the experiment, which further negated the disruption by the machine, reducing the severity of the fall. This is demonstrated through the human-robot interaction force in Fig. 15, in which the force magnitude of some fall trials could be comparable to the ones of normal walking. In order to cover more realistic fall scenarios, which are likely to be more severe for the case of impaired individuals, a second experiment was conducted. Healthy subjects were invited to perform self-induced falls, in which they were instructed not to regain balance by themselves unless necessary. Only force sensor is used for the evaluation in Fall Experiment 2 due to its higher sensitivity and specificity found in Fall Experiment 1. The threshold to indicate a loss of balance was further optimized for Fall Experiment 2. The instability detection algorithm has identified approximately 94% of self-induced falls with a false alarm rate of 0%, yielding a G-Mean score of 0.973. The details are tabulated in Table 5.

**Table 5:** The results of instability detection method in Fall Experiment 2

	NW_SW	NW_C	FS_RO	FS_W	Sensitivity	Specificity	G-Mean
Detection Rate	0.000	0.000	0.958	0.936	0.947	1.000	0.973

The algorithm compares the force sensor value to a predefined threshold to identify loss of balance. Detection rate $= \frac{\text {number of detected trials}}{\text {number of trials}}$

## Discussion

When being connected to MRBA, both healthy subjects and the SCI subject experienced a change in the gait parameters, in which their walking speed and step size decreased. Their lower limb joint kinematics also changed significantly from the interaction with MRBA. The effects of overground gait rehabilitation and assistive devices on human gait were well-documented in the literature. Burgess et al. have evaluated KineAssist with healthy and post-stroke subjects [26]. Similar to the case of MRBA, the healthy subject walking speed decreased from their regular $1.2\pm 0.2$ m/s to $0.7\pm 0.2$ m/s when using the device. Their step length also decreased as more body weight support was applied to the subjects. The lesser gait speed reduction by MRBA than KineAssist may be attributed to the intrinsic transparency of MRBA pelvic interface; the transparency of KineAssist heavily depends on their control algorithm. Stroke patients experienced a similar change in gait with their speed and step length decreasing from $0.8\pm 0.3$ m/s to $0.4\pm 0.1$ m/s and $0.5\pm 0.08$ m to $0.3\pm 0.08$m, respectively. The NUS Walker was evaluated by Mun et al. by studying the pelvis motion of healthy subjects walking with and without the device [27]. The mean velocity decreased from 1.0 m/s to 0.5 m/s and the rotation of the pelvis about the vertical axis was also reduced. The other study found significant reduction in ankle plantarflexion, knee flexion and hip flexion, as well as decrease in step length and step width [7]; similar observations were discovered for MRBA except for step width reduction. By providing support to the users, NaTUre-gaits has decreased muscle activation, produced more consistent walking patterns and offloaded the user’s weight [28, 29]. The experimental results concur with the literature findings in which that the natural gait speed of both healthy subjects and patients was reduced when interacting with these devices. This is because the subject’s body weight is supported, forcing them to alter their gait to minimize the level of energy expenditure [30]. Such change in gait patterns is not detrimental as it is a natural occurrence due to the human–machine interaction when a gait assistive device restraints the user’s movement, which causes the user to take smaller steps and hence, smaller joint angle range of motion. For slow ambulators with severely impaired balance, the safety provided by the assistive robot could potentially outweigh the forced reduction in gait speeds.

In Fall Experiment 1, it was found that the instability caused by the FIMP-induced falls could be insignificant due to the high balance capability of the test subjects. As a result, when the instability detection algorithm was optimized to identify the FIMP-induced falls, the detection threshold was set low in order to detect more falls. However, this led to significant false alarms yet sub-optimal fall detection accuracy. The best sensory data was the force sensor data, which identified the loss of balance in more than two-thirds of the FIMP-induced falls with a false positive rate of around 20%. The IMU data yielded poor performance in instability detection, which may be caused by the measurement noise and the minimal change in sensor value during a loss of balance.

To create more realistic fall scenarios which could be severe for impaired individuals, a second experiment was conducted with self-induced falls, in which the subjects simulate falls as instructed without trying to recover their balance. Due to the severe loss of balance, the performance of the algorithm improved significantly as the difference between the loss of balance and regular walking widened. The threshold to detect instability was increased such that it exceeded the human-robot interaction force of normal walking yet was still low enough to cover most severe falls. Nevertheless, as the force sensors were implemented such that they only capture forces applied along the vertical axis, there were some loss of balance scenarios in which the current setup was unable to detect. Such cases were considered to be minority based on the experimental observation as most falls would cause the users to fall downwards when the subjects’ lower limbs gave way during a fall.

The two fall experiments show that there is room for improvement in the current instability detection algorithm, in which it should identify most if not all the loss of balance trials while recognizing the regular gaits as non-detrimental. In the study of instability detection, it is always hard to define how stringent the criteria should be in identifying the loss of balance. If the criteria are too loose, while the system can capture all forms of loss of balance, it will create a lot of false alarms too. On the other side of the spectrum, the safety of the person will be severely compromised if their instability goes unnoticed. Other learning-based methods should be explored to improve the detection quality. The learning-based methods can be leveraged to analyze the balance state of the user based on multiple sensory data.

**Fig. 16:**
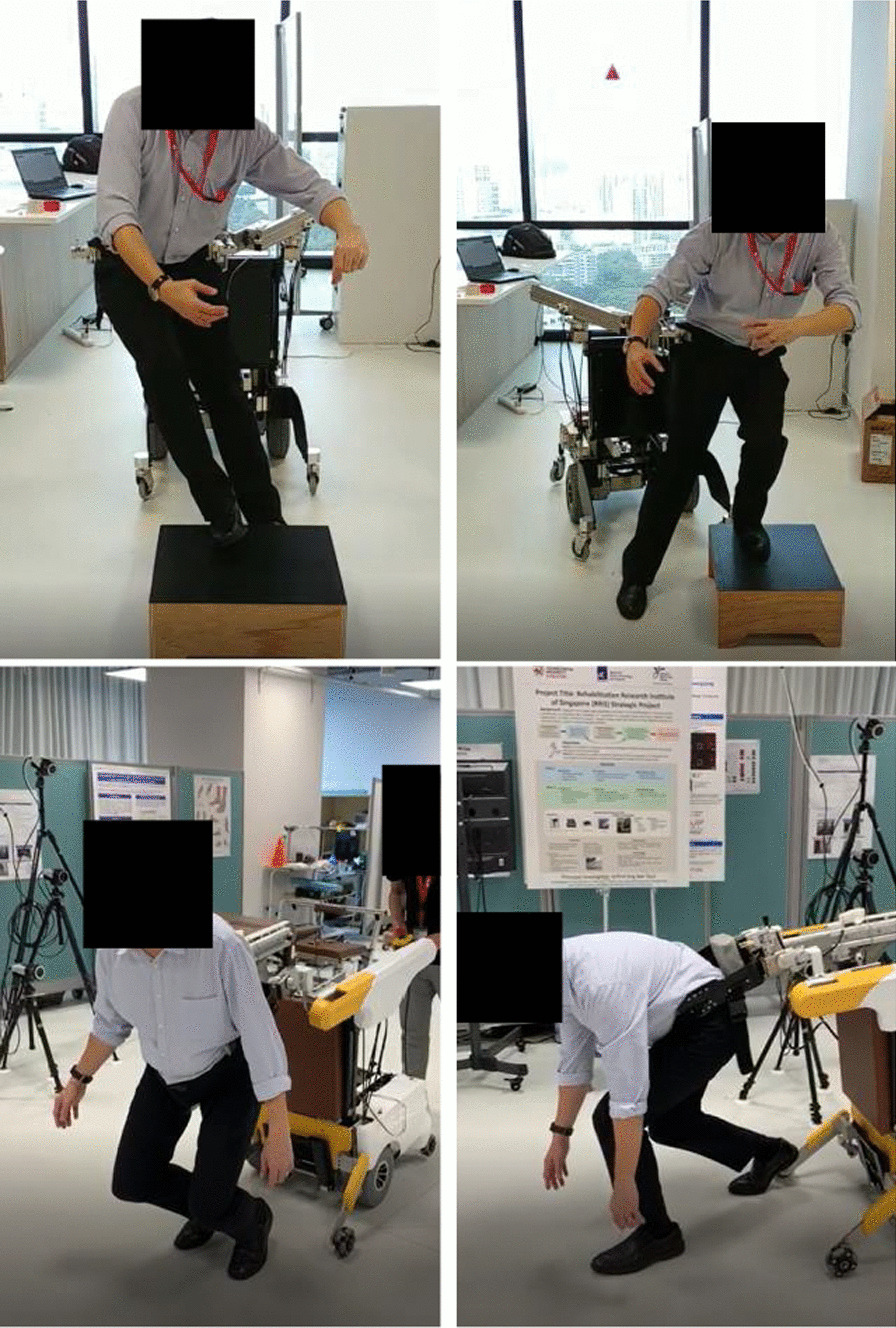
A physiotherapist evaluating MRBA with different types of loss of balance motion. Top: tilting to one side while standing. Bottom: simulating a fall when walking with MRBA

One limitation in the instability detection study is that all the subjects were healthy young adults. The first Fall Experiment showed that the subjects’ stability was only mildly affected by FIMP due to the high balance capability of the young subjects, which allowed them to recover easily from the perturbation of FIMP. The mitigated loss of balance might be one of the causes for the poor detection rate of the FIMP-trials. The findings led to Fall Experiment 2, which required the subjects to simulate falls themselves without recovery. Nevertheless, the limitation of simulated falls by healthy young population may make the experimental results on the instability detection algorithm questionable. Thus, the algorithms were further assessed by physiotherapists who demonstrated various types of falls that commonly occur among the patient population. For example, a physiotherapist has demonstrated that MRBA was able to prevent him from falling when he tilted to different directions when standing; the robot also arrested falls occurred during walking. Figure 16 shows the physiotherapist being supported by MRBA during prototype testing. In summary, the physiotherapists have verified that the robot performance is adequate for patient fall prevention.

When evaluating the instability detection algorithm optimized based on the healthy subject data on the SCI normal walking data, the algorithm produced false alarms for all the trials. It was observed that the subject relied on the body weight support provided by MRBA, causing the force sensors to measure much higher readings than the healthy subject walking. The gait patterns also differ between healthy users and impaired users. The observation suggests that patients’ data is critical in fine-tuning the instability detection algorithm for the actual application. Nevertheless, collecting fall data from individuals with balance impairment is a challenging hurdle to be overcome.

## Conclusions

There are currently no commercially available robotic technological solutions for fall intervention in the home environment. The available solutions for balance assistance are designed as rehabilitation systems with large footprint and low maneuverability, making them undesirable to be used at home.

The developed Mobile Robotic Balance Assistant (MRBA) seeks to solve this limitation. Unlike the available solutions, MRBA has a small footprint. It also comes with an intrinsically compliant pelvic interface that eliminates the need of a feedback algorithm to control the motion of the interface. The robot follows the user around when they are performing regular activities of daily living to provide balance assistance. In an experiment involving regular walking and self-simulated falls, the instability detection algorithm can identify more than 93% of the falls with 0% false positive rate. When the algorithm detects that the user has lost their balance, the robot activates its fall intervention system to secure the user in place. Combining the assistive technology to a regular powered wheelchair allows the user to sit down when they are fatigue or need to commute quickly. The transition between the sitting and standing posture is physically supported by the system, ensuring the safety of the user. The study on the able-bodied population and balance impaired subjects shows that MRBA has caused significant reduction in self-selected walking speed, which was also observed in other existing gait assistive technologies. The change in gait speed could be a trade-off for reduction in fall risks. We hope that the device can encourage the balance impaired population to walk more and engage in activities of daily living, hence promoting the recovery of their balance capability and improving their quality of life.

### Future work

In future research, a more thorough evaluation with patients is needed to understand how the robot affects pathological gait. The patient data can also help to devise the proper instability detection methods for the target audience. The effectiveness of MRBA can be bench-marked against manual human assistance and common walking aids.

Furthermore, there is room for improvement in the current design.

Firstly, the current user following algorithm implements a simple proportional control that regulates the distance between the robot and the user. While it is sufficient to achieve the intended functionality, it still significantly alters their walking patterns. Moreover, different users usually demand different control parameters, which are costly to be customized for each individual. A more intelligent approach may be necessary to better cater to the needs of different users. The method should also understand the user intention accurately for smoother human-robot interaction.

Secondly, the fall detection algorithm needs further improvement. Solely evaluating the force sensor data against a fixed threshold creates false alarms and miss-detection even for the case of healthy individuals. Moreover, due to patient’s weakened ability, they require more body weight support from the system, especially when they are exhausted after prolonged physical activities. The increased in exerted force causes the fall detection algorithm to be triggered more frequently, significantly hampering the usability of the device. Depending on how the user loses balance, the force sensors may not detect the instability if the person falls in a direction that does not exert forces onto the sensors, such as the cases in Fall Experiment 2. Hence, the fall detection algorithm has to examine more sensor data when evaluating the user stability, as well as to explore machine learning algorithms to achieve better sensitivity and specificity.

Lastly, in the current settings, when a loss of balance is detected, MRBA immediately stops and locks the pelvic interface to allow the user to recover balance by themselves or call for human assistance. While this is adequate for cases in which an inevitable fall occurs, more subtle assistance is required to further enhance the robot capability as an assistive device. Under most scenarios, the users do not completely lose their balance; they may stumble a little or sway in different directions due to poor balance capability. A light intervention force from the robot to correct the instability is more appropriate than a complete stop of the robot because it allows the user to train their balance reflexes while mitigating the implications of false alarms. To achieve this, the user’s instability status has to be accurately quantified such that the variable stiffness actuators can be programmed to deliver the appropriate assistive forces.

## Supplementary information

**Additional file 1. **MRBA Demonstration Video. The video shows the usage of MRBA on different types of scenarios, namely regular walking, sit-to-stand and fall intervention.

**Additional file 2.** MRBA Sensor Data (Comparison between Normal Walking and Turning). The plots of the data trend of body joint angles and their SPM1D results of straight walking (NW SW) and turning (NW C) with healthy subjects. The blue line and the orange line represent the data of NW SW and NW C, respectively. The dotted line represents the end of the stance phase and the beginning of the swing phase. In each subfigure, the first row shows the mean of the data with its standard deviation as shaded region. The second row shows the F-values compared against the threshold. Statistical results greater than the threshold indicate a statistically significant difference between the two groups.

**Additional file 3. **MRBA Sensor Data (Comparison between Normal Walking and Mid-Swing Trip). The plots of the data trend of body joint angles and their SPM1D results of straight walking (NW SW) and turning (FE MS) with healthy subjects. The blue line and the orange line represent the data of NW SW and FE MS), respectively. The dotted line represents the end of the stance phase and the beginning of the swing phase. In each subfigure, the first row shows the mean of the data with its standard deviation as shaded region. The second row shows the F-values compared against the threshold. Statistical results greater than the threshold indicate a statistically significant difference between the two groups.

**Additional file 4. **MRBA Sensor Data (Comparison between Normal Walking and Slip). The plots of the data trend of body joint angles and their SPM1D results of straight walking (NW SW) and turning (FE SL) with healthy subjects. The blue line and the orange line represent the data of NW SW and FE SL, respectively. The dotted line represents the end of the stance phase and the beginning of the swing phase. In each subfigure, the first row shows the mean of the data with its standard deviation as shaded region. The second row shows the F-values compared against the threshold. Statistical results greater than the threshold indicate a statistically significant difference between the two groups.

**Additional file 5. **MRBA S MRBA Sensor Data (Comparison between Normal Walking and Knee Buckling). The plots of the data trend of body joint angles and their SPM1D results of straight walking (NW SW) and turning (FE KB) with healthy subjects. The blue line and the orange line represent the data of NW SW and FE KB, respectively. The dotted line represents the end of the stance phase and the beginning of the swing phase. In each subfigure, the first row shows the mean of the data with its standard deviation as shaded region. The second row shows the F-values compared against the threshold. Statistical results greater than the threshold indicate a statistically significant difference between the two groups.

## Data Availability

The datasets generated and/or analyzed during the current study are available from the corresponding author upon reasonable request.

## Abbreviations

ADL: Activity of daily living
ANOVA: Analysis of variance
AP: Anterior–posterior
CoM: Center of mass
FE_KB: Knee buckling trial
FE_MS: Mid swing trip trial
FE_SL: Slip trial
FIMP: Fall Inducing Movable Platform
FS_RO: Reaching out fall trial
FS_W: Walking and fall trial
FW: Free walking
GYRO: Gyroscope reading
IMU: Inertia measurement unit
ML: Medial–lateral
MRBA: Mobile Robotic Balance Assistant
NW_C: Walking in circles with MRBA
NW_SW: Straight walking with MRBA
RGB-D: Red-blue-green-depth
SCI: Spinal cord injury
SPM: Statistical parametric mapping
