# Supplementary material for: Mobile Robotic Balance Assistant (MRBA): a gait assistive and fall intervention robot for daily living
Source: J Neuroeng Rehabil. 2023 Mar 1;20:29. doi: 10.1186/s12984-023-01149-0 (PMC9979429; doi:10.1186/s12984-023-01149-0)
Supplement: Supplementary file 5 — Additional file 5. MRBA S MRBA Sensor Data (Comparison between Normal Walking and Knee Buckling). The plots of the data trend of body joint angles and their SPM1D results of straight walking (NW SW) and turning (FE KB) with healthy subjects. The blue line and the orange line represent the data of NW SW and FE KB, respectively. The dotted line represents the end of the stance phase and the beginning of the swing phase. In each subfigure, the first row shows the mean of the data with its standard deviation as shaded region. The second row shows the F-values compared against the threshold. Statistical results greater than the threshold indicate a statistically significant difference between the two groups. [file 12984_2023_1149_MOESM5_ESM.pdf]

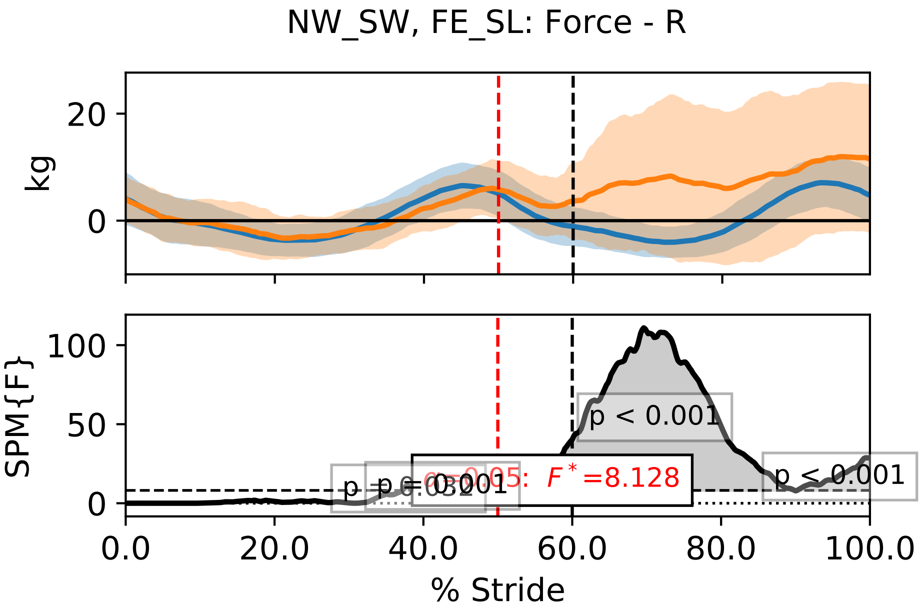

NW_SW, FE_SL: Force - R
kg
20
0
SPM{F}
100
50
0
p < 0.001
p < 0.001
F*=8.128
0.0
20.0
40.0
60.0
80.0
100.0
% Stride

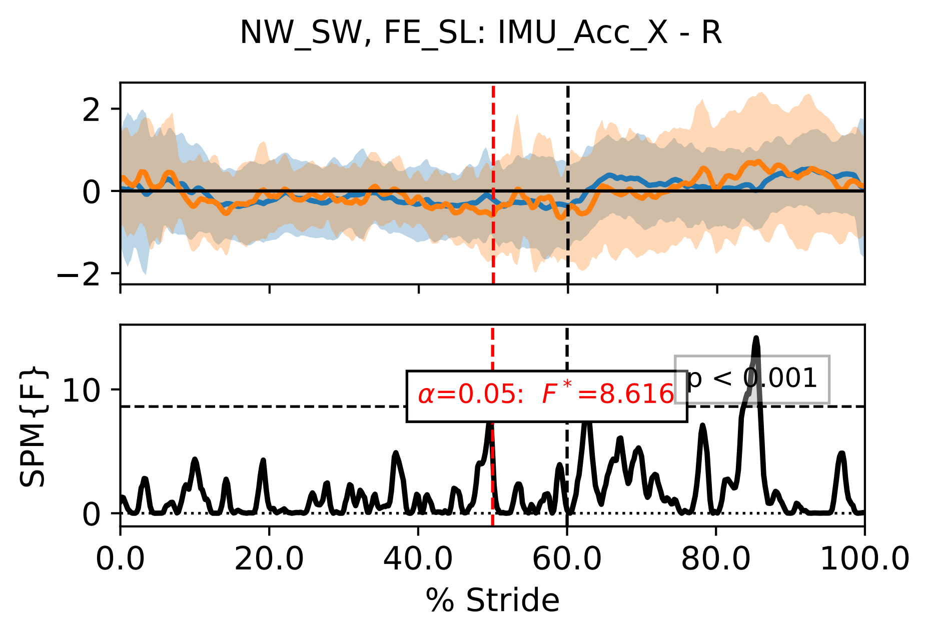

NW_SW, FE_SL: IMU_Acc_X - R
2
0
−2
SPM{F}
10
0
α=0.05: F*=8.616
p < 0.001
0.0
20.0
40.0
60.0
80.0
100.0
% Stride

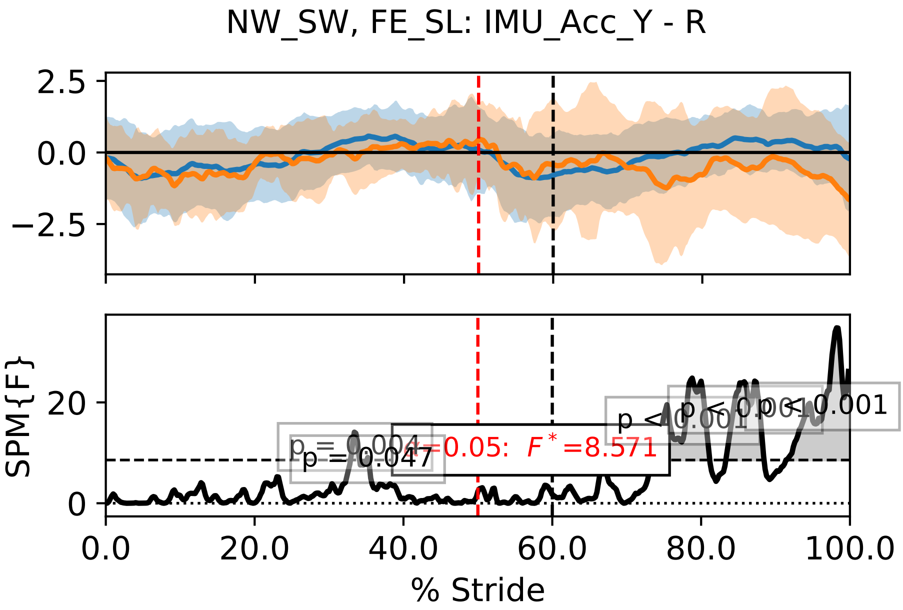

NW_SW, FE_SL: IMU_Acc_Y - R
2.5
0.0
−2.5
SPM{F}
20
0
p = 0.004
p = 0.047
α=0.05: F^*=8.571
p < 0.001
p < 0.001
p < 0.001
0.0
20.0
40.0
60.0
80.0
100.0
% Stride

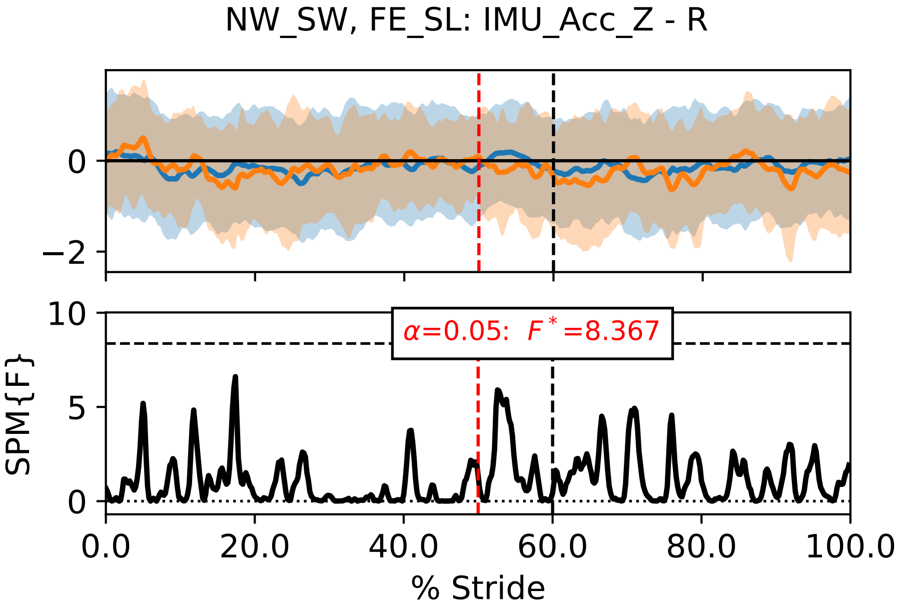

NW_SW, FE_SL: IMU_Acc_Z - R
0
−2
10
5
0
SPM{F}
α=0.05: F*=8.367
0.0
20.0
40.0
60.0
80.0
100.0
% Stride

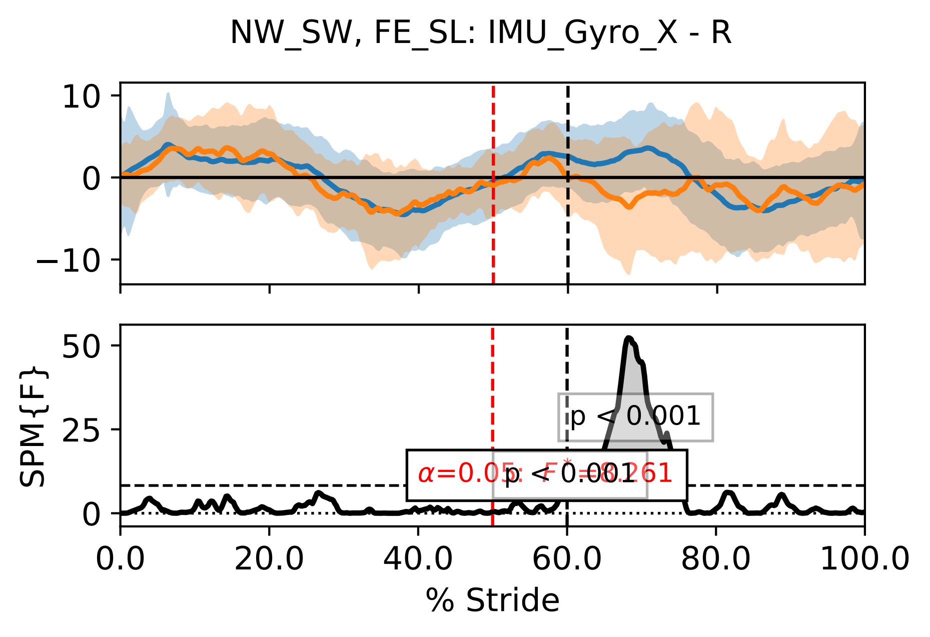

NW_SW, FE_SL: IMU_Gyro_X - R
10
0
−10
50
25
0
SPM{F}
p < 0.001
α=0.05: F*=8.261
p < 0.001
0.0
20.0
40.0
60.0
80.0
100.0
% Stride

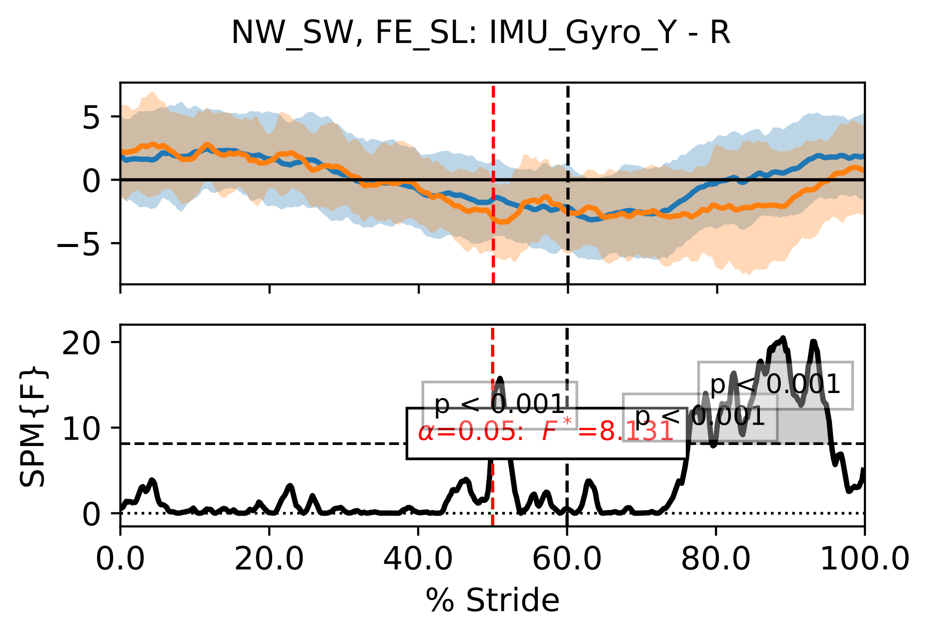
NW_SW, FE_SL: IMU_Gyro_Y - R
5
0
−5
20
10
0
SPM{F}
p < 0.001
p < 0.001
p < 0.001
α=0.05: F* =8.131
0.0
20.0
40.0
60.0
80.0
100.0
% Stride

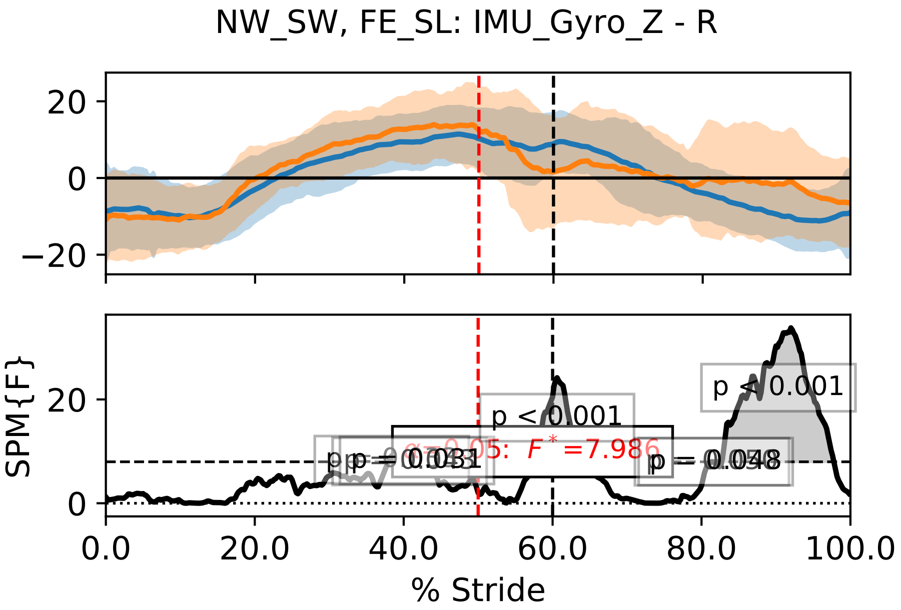
NW_SW, FE_SL: IMU_Gyro_Z - R
SPM{F}
% Stride
p < 0.001
p < 0.001
α=0.05: F* =7.986
